# University of Louisville

**Published:** 1851-09

**Authors:** 


UNIVERSITY OF LOUISVILLE.

The attention of medical students is invited to the preliminary course of Lectures announced in this institution.

